# Supplementary material for: Microbiota-dependent indole production stimulates the development of collagen-induced arthritis in mice
Source: J Clin Invest. 2023 Dec 19;134(4):e167671. doi: 10.1172/JCI167671 (PMC10866668; doi:10.1172/JCI167671)
Supplement: Supplemental data [file jci-134-167671-s008.pdf]

Supplemental Methods

Animal studies. *Collagen-induced arthritis.* Six-week-old male DBA/1 mice (The Jackson Laboratory or Envigo) were injected intradermally with 200μg bovine CII (Elastin) emulsified in 50μl complete Freund's adjuvant (CFA, Sigma) on days 0 and 21. Disease severity was measured as the sum of the clinical scores for each of the animal's four paws, where 0 = normal, 1 = one swollen digit, 2 = two swollen digits, 3 = three swollen digits, and 4 = entire paw swollen with ankylosis. Mice were euthanized at day 14, 21, or at the plateau of CIA severity (day 35-50) and feces, serum, and tissues were collected.

Antibiotic treatment. Antibiotics were administered as previously described (1). Briefly, ampicillin (RPI, 1g/L) neomycin (RPI, 1g/L), vancomycin (Alfa Aesar, 0.5g/L), metronidazole (RPI, 0.5g/L), and grape-flavored Kool-Aid (20g/L) (Kraft Foods, to encourage consumption) were given in drinking water to mice beginning on day 21 through the end of the study. Kool-Aid alone was provided to the control group.

Dietary intervention. On CIA day -1, mice were given either Amino Acid chow (AA; Envigo, TD.01084, 0.18% L-Trp) or Trp Deficient Diet VI (Envigo, TD.130674). "Trp-Sufficient" (TS) mice were maintained on the AA diet for the duration of the experiment. "Trp-Low" (TL) mice were alternated between 5 days of Trp Deficient Diet VI and 2 days of Amino Acid diet for a cumulative 0.05% Trp-low diet. Indole (Acros Organics, 0.1mg/ml) was given in drinking water to mice beginning on day 21 through the end of the study. Alternatively, 200μl of 10mM indole in water was administered by oral gavage every other day beginning on day 0.

E. coli *colonization.* 6-week old germ-free DBA/1 mice were maintained on standard rodent show (Envigo 2020SX) containing 0.2% L-Trp. On day -7, mice received 10^8 CFU *E. coli* BW25113 *ΔtnaA* or *E. coli* BW25113 *ΔbcsQ*. *ΔbcsQ* was selected as the isogenic control because *bcsQ* is a pseudogene in *E. coli* BW25113 and should have little to no phenotype in these studies. CIA was induced on day 0 after *E. coli* colonization was established. *E. coli* strains were obtained from GE Dharmacon (Lafayette, CO).

Microbiome Analysis. Total genomic DNA was extracted using the QIAamp PowerFecal DNA kit (Qiagen Inc, Carlsbad, CA), which employs chemical and mechanical disruption (Roche MagNA Lyser) of biomass. PCR

amplicons were generated using barcoded (2) primers targeting the V3V4 variable region of the 16S rRNA gene (338F: 5'ACTCCTACGGGAGGCAGCAG and 806R: 5' GGACTACHVGGGTWTCTAAT) (3, 4). PCR products were normalized using a SequalPrep™ kit (Invitrogen, Carlsbad, CA) and then pooled. The amplicon pool was partially lyophilized to reduce its volume, purified and concentrated using a DNA Clean and Concentrator Kit (Zymo, Irvine, CA), and then quantified using a Qubit Fluorometer 2.0 (Invitrogen, Carlsbad, CA). The pool was diluted to 4nM and denatured with 0.2 N NaOH at room temperature. The denatured DNA was diluted to 15pM and spiked with 25% of the Illumina PhiX control DNA prior to loading the sequencer. Paired-end sequencing was performed on the Illumina MiSeq platform with versions v2.4 of the MiSeq Control Software and of MiSeq Reporter, using a 600 cycle version 3 reagent kit.

Paired-end sequences were sorted by sample via barcodes in the paired reads with a Python script (5, 6). The paired reads were assembled using phrap (7, 8) and pairs that did not assemble were discarded. Assembled sequence ends were trimmed over a moving window of 5 nucleotides until average quality met or exceeded 20. Trimmed sequences with more than 1 ambiguity or shorter than 250 nt were discarded. Potential chimeras identified with Uchime (usearch6.0.203_i86linux32)(9) using the Schloss (10) Silva reference sequences were removed from subsequent analyses. Assembled sequences were aligned and classified with SINA (1.3.0-r23838) (11) using the 418,497 bacterial sequences in Silva 115NR99 (12) as reference configured to yield the Silva taxonomy; taxonomic assignments used the lowest-common-ancestor approach with default SINA settings. Closed-reference, operational taxonomic units were produced by binning sequences with identical Silva/SINA LCA assignments. Taxa with >0.01% abundance in any sample and observed in >5% of the samples were included in further analyses. All samples had a Good's coverage index >99%, indicating excellent depth of sequencing coverage.

The software packages R (v3.6.3) (13) and Explicet (v2.10.5) (14) were used to analyze and visualize data. Alpha-diversity indices (i.e., Chao1, Shannon H, Shannon H/Hmax) were evaluated between groups by ANOVA. Differences in overall microbiota composition (i.e., beta-diversity) were assessed through permutational ANOVA (PERMANOVA) with the Aitchison dissimilarity index applied to sequence count data (15, 16). Principal coordinates analysis (PCoA) was carried out using Aitchison dissimilarities and the *wcmdscale* function in the *vegan* R package.(16) Individual taxa differing between treatment groups were identified using the ANOVA-like differential expression (ALDEx2) R package (17, 18). The distribution of taxa in each sequence library was

estimated through 1000 Dirichlet Monte Carlo re-samplings of sequence count data. To account for the compositional nature of microbiome sequence data, datasets were then subjected to a center log-ratio transformation with all features used as the denominator. P-values were adjusted for multiple comparisons using the false discovery rate method (19). Effect size plots are derived from the outputs of ALDEx2 and represent the median effect sizes, calculated as the median between-group difference in CLR values between groups divided by the largest within-group difference in CLR values (17, 18).

Metabolomics. Cecal tip (tissue and contents, 30-100 mg) were harvested at day 35, flash-frozen, and stored at -80°C. Metabolomic analyses were performed via one of three methods as follows:

HPLC: Metabolites were extracted as previously described (20) with minor variations. Briefly, cecal tissue and contents were reconstituted in 200μl of HPLC-grade 80% methanol, sonicated for 3 x 3 second pulses (BioLogics Inc., 150 V/T Ultrasonic Homogenizer, power output ~20%), and then centrifuged at 12,000g for 5 minutes. The supernatant was saved and the extraction was repeated for a total of three rounds, producing a total of 600μl of extract. Samples were filtered through 5kDa spin columns (Amicon) and metabolites analyzed by HPLC. Analyses were performed on an Agilent Technologies 1260 Infinity HPLC using a Sepax Br-C18 column (120 Å, 4.6 x 250 mm). Mobile phase A, HPLC-grade water pH 7.0; mobile phase B, HPLC-grade acetonitrile; column temperature at 30 °C and flow rate of 1 ml/min. Chromatographic separation of the metabolites was performed using a gradient of 10% to 90% B in 30 min followed by washing and equilibration periods at the end of each run. The indole derivatives were detected by absorption at 280 nm and their absorbance spectra and retention times were confirmed by co-injection with authentic standards. Area under the curve (AUC) was calculated for each metabolite and normalized to starting sample weight.

HPLC-MS: Indole derivatives were quantified in mouse cecal samples using reversed-phase high-performance liquid chromatography with electrochemical coulometric array detection (EC-HPLC; CoulArray, Thermo Scientific, Waltham, MA). Cecal samples were extracted in 80% methanol and protein precipitate was removed by centrifugation at 15,000 x g. Separation was achieved using an Acclaim Polar Advantage II C18 column (Thermo, Waltham, MA) at a flow rate of 1 ml/min on a gradient of 10% to 55% acetonitrile in 50 mM sodium phosphate buffer, pH=3, containing 0.42 mM octanesulphonic acid as an ion-pairing agent. Calibration curves

were composed by performing linear regression analysis of the peak area versus the analyte concentration. The data were quantified using the peak area in comparison to standards.

LC-MS/MS: cecal metabolites were analyzed by LC-MS/MS as described previously (21).

Detection of serum or supernatant cytokines. A multianalyte ELISA (MesoScale Diagnostics) was used to measure the levels of TNF (lower limit of detection, LLOD, 1.3 pg/ml), IL-1β (LLOD 3.1 pg/ml), IL-6 (LLOD 4.8 pg/ml), IL-17A (LLOD 0.3 pg/ml), IL-10 (LLOD 3.8 pg/ml), IFNγ (LLOD 0.16 pg/ml), IL-21 (LLOD 6.5pg/ml), IL-22 (LLOD 1.2 pg/ml), IL-23 (LLOD 4.9 pg/ml), and GM-CSF (LLOD 0.16pg/ml) in either undiluted serum or supernatant, according to the manufacturer's protocol.

Detection of CII-specific antibodies. Type II collagen-specific antibodies were detected in mouse serum at CIA day 35 using previously published methods (22). Briefly, 96-well plates (Nunc MaxiSorp) were coated overnight at 4C with 5μg/ml ELISA-grade bovine CII (Chondrex), washed 3x with PBS + 0.05% Tween-20, and blocked for 4hr at 4C with 1% BSA in PBS. Serum samples were added to the wells at a dilution of 1:40,000 and incubated overnight at 4C with rocking. The plates were then washed, and horseradish peroxidase (HRP)-conjugated goat anti-mouse IgG, IgG1, IgG2a, or IgG2b (SouthernBiotech, Supplemental Table 4) were added to the wells at a dilution of 1:10,000 for 4 hours at 4C. Following washing, 1X TMB ELISA substrate solution (BD Biosciences) was added to the wells and the plates were developed at room temperature. The reaction was stopped with 2N H_2SO_4 and read at 450nm and 570nm wavelengths. Pooled serum from mice with severe CIA was used to generate a standard curve in which the top standard was diluted 1:1000 (1unit/ml), followed by 2-fold serial dilutions.

C3 activation ELISA. Complement binding to CII-specific antibodies was assessed using previously published methods (1). Ninety-six–well plates were coated overnight at 4°C with 5 μg/ml ELISA-grade mouse CII. The ELISA plates were then washed three times with 0.1% BSA + 0.05% Tween-20 in 1X PBS and blocked with 1% BSA in 1× PBS for 4 hours at 4°C. Serum samples were diluted 1:10,000 in 1× PBS. Samples were added to wells and incubated overnight at 4°C. The next day, wells were washed 5 times with 1× PBS + 0.05% Tween-

20, then incubated with 15% normal mouse serum diluted in Dulbecco's PBS + 0.9 mM $CaCl_2$ + 0.5 mM $MgCl_2$ for 30 minutes at 37°C. The plates were then washed 5 times with 1× PBS and incubated with HRP-conjugated goat IgG to mouse C3 (Cappel/MP Biomedicals, Supplemental Table 4) in 1:2,500 dilution for 1 hour at room temperature with rocker shaking. The plates were washed 5 times with 1× PBS and developed with 1× TMB ELISA substrate solution for 10 minutes. The reaction was then stopped with H_2SO_4 and read at 450 nm.

Glycosylation studies. Serum total IgG was purified by using Pierce Protein G Agarose (ThermoFisher Scientific) following the manufacturer's instructions. CII-specific antibodies were purified from serum as previously described (23) by coupling bovine CII (Chondrex) to CNBr-activated Sepharose 4B beads. To concentrate the eluted total IgGs, 3-kd Ultra-0.5 ml centrifugal filter units (Amicon) were used. Total N-linked glycan was released from glycoproteins using PNGase F (New England Biolabs) according to the manufacturer's instructions. Deglycosylation reactions were carried out at 37°C overnight to ensure effective release of glycans. Glycans were purified from the reaction using GlykoClean G Cartridges (Prozyme), dried, and fluorescence labeled with 2-aminobenzamide (Sigma-Aldrich). Labeled glycans were cleaned with GlykoClean S-plus Cartridges (Prozyme), dried, and subjected to high-performance liquid chromatography analysis. Glycan samples were dissolved in 25% 100 mM ammonium formate (pH 4.5) and 75% acetonitrile then separated using an Agilent 1260 Infinity Quaternary LC system outfitted with a 2.1 × 150 mm AdvanceBio Glycan Mapping column with 2.7 μm superficially porous particles and a fluorescence detector. Resulting peaks were analyzed in OpenLAB software (Agilent) and assigned glycoforms by comparing peaks of commercially available human IgG N-linked glycan library.

Flow analysis. Tissues were harvested from mice with CIA at day 21 or 35, homogenized in RPMI media, and passed through a 70 micron cell strainer. Red blood cells were lysed using Red Blood Cell Lysis buffer (eBioscience), and resuspended in FACS buffer (5% FBS in PBS) for surface staining. For intracellular staining, cells were fixed and permeabilized using the FoxP3/Transcription Factor Staining Buffer Kit (Tonbo). All antibodies and clones used are listed in Supplemental Table 3. Flow cytometric analysis was performed at the Barbara Davis Center BioResource Service Center, and analysis was performed using FlowJov10 software.

Splenocyte re-stimulation. Splenocytes were harvested as described above and re-stimulated with UV-crosslinked bovine Type II Collagen at a final concentration of 500ug/ml for 72 hours. Alternatively, $5x10^5$ splenocytes were stimulated with 2µl pre-washed CD3/CD28 Dynabeads. Supernatants were stored at -20C and were analyzed by multiplex immunoassay (MSD) as described above.

Histopathology. Mouse paws were removed at mid-limb and fixed in 10% paraformaldehyde. The bones were decalcified in 10% formic acid for 1 week and then embedded in paraffin. Sections of 5µm were cut from paraffin embedded tissues and stained with H&E. Pathology was assessed in a blinded manner for inflammation, pannus formation, and bone erosion. Pannus scoring criteria: 0 = no areas affected; 0.5=Very minimal, marginal zone only, less than 1% of area at risk affected; 1=Minimal infiltration of pannus in cartilage and subchondral bone, marginal zones mainly. Approximately 1-10% of area at risk affected; 2=Mild infiltration with marginal zone destruction of hard tissue in affected joints, 11-25% of area at risk affected; 3=Moderate infiltration with moderate hard tissue destruction in affected joints, 26-50% of area at risk affected; 4=Marked infiltration with marked destruction of joint architecture, affecting most joints, 51-75% of area at risk affected; 5=Severe infiltration associated with total or near total destruction of joint architecture, affects all joints, greater than 75% of area at risk affected. Bone resorption scoring criteria: 0.5=Very minimal resorption affects only marginal zones; 1=Minimal approximately 1-10% of area at risk of subchondral bone affected; 2=Mild, more numerous areas of resorption, approximately 11-25% of total area at risk of subchondral bone affected; 3=Moderate, obvious resorption of subchondral bone resulting in approximately 26-50% of area at risk of subchondral bone affected; 4=Marked, very obvious resorption of subchondral bone resulting in approximately 51-75% of area at risk of subchondral bone affected; 5=Severe, distortion of entire joint due to destruction approximately 76-100% of area at risk of subchondral bone affected. Inflammation scoring criteria: 0=no inflammatory infiltrate; 1=mild cellular infiltrate into joint and synovium; 2=enhanced cellular infiltrates, increased cell density throughout the joints, some joints affected; 3=maximal inflammation, high cell density, all joints affected.

Complement C3 Immunohistochemistry. Paraffin-embedded tissue slides were assessed for C3 complement deposition in the joints as described previously (24) using Goat Anti-Mouse Complement C3 (MP Biomedical, Supplemental Table 4). Complement deposition in each of the four paws was scored by a blinded observer from

0 to 3 (0=no staining, 1=mild staining, 2=moderate staining, 3=intense staining), and the average score across the four paws was plotted.

Collection and Isolation of Human LPMC. Colon tissue samples (N=5) were procured from the Program for Individuals with an Elevated Risk of Spondyloarthritis (PIERS) Registry. Healthy tissue was obtained from patients undergoing bowel surgery and would otherwise be discarded. These patients had no existing rheumatic disease or a history of Inflammatory Bowel Disease, HIV-1 infection, current treatment with immunosuppressive drugs, or recent chemotherapy (within 8 weeks). All patients undergoing surgery consented to the use of discarded tissue for research purposes. Protected patient information was de-identified to the laboratory investigators.

Lamina propria mononuclear cells (LPMC) were isolated from tissue samples as previously detailed (25-27). Briefly, tissue specimens were trimmed of muscle and fat and treated with DL-Dithiothreitol (DTT; 1.67mM; Sigma-Aldrich) to remove additional mucus. The epithelial layer was subsequently removed with 1mM EDTA (Sigma-Aldrich) and the remaining tissue treated with collagenase D (0.5mg/ml, Roche Diagnostics). All released LPMCs were cryopreserved and stored in liquid nitrogen.

Cryopreserved LPMCs were thawed and stimulated at 37C with 1mM indole for 4 hours. CD3+ T cells and CD19+ B cells were flow sorted using a FACSAriaIII cell sorter. Total RNA was extracted using an RNeasy kit (Qiagen) according to the manufacturer's protocol. Libraries were constructed as previously described using the Next Ultra II directional RNA library prep kit with rRNA depletion (28). Bulk RNA sequencing was performed on an Illumina MiSeq platform at the University of Colorado Genomics core. RNA-Sequencing workflow was implemented through the Bioconductor differential expression pipeline (29, 30). Salmon (31) was used for transcript quantification with GC bias correction, using a human transcript reference index from GENCODE (release 38) and no decoy sequences (32). Tximeta (33) was used for importing transcripts which were then analyzed for differential expression with DESeq2 (34). Ingenuity Pathway Analysis as used to identify differentially expressed pathways. Differentially expressed pathways were defined as those with a p-value <0.05 and a z-score >2.

Supplementary References

1. Jubair WK, Hendrickson JD, Severs EL, Schulz HM, Adhikari S, Ir D, et al. Modulation of Inflammatory Arthritis in Mice by Gut Microbiota Through Mucosal Inflammation and Autoantibody Generation. *Arthritis & Rheumatology.* 2018;70(8):1220-33.
2. Frank DN. BARCRAWL and BARTAB: software tools for the design and implementation of barcoded primers for highly multiplexed DNA sequencing. *BMC bioinformatics.* 2009;10:362.
3. Lane DJ, Pace B, Olsen GJ, Stahl DA, Sogin ML, and Pace NR. Rapid determination of 16S ribosomal RNA sequences for phylogenetic analyses. *Proc Natl Acad Sci U S A.* 1985;82(20):6955-9.
4. Weisburg WG, Barns SM, Pelletier DA, and Lane DJ. 16S ribosomal DNA amplification for phylogenetic study. *J Bacteriol.* 1991;173(2):697-703.
5. Chriswell ME, Lefferts AR, Clay MR, Hsu AR, Seifert J, Feser ML, et al. Clonal IgA and IgG autoantibodies from individuals at risk for rheumatoid arthritis identify an arthritogenic strain of Subdoligranulum. *Sci Transl Med.* 2022;14(668):eabn5166.
6. Frank DN, Qiu Y, Cao Y, Zhang S, Lu L, Kofonow JM, et al. A dysbiotic microbiome promotes head and neck squamous cell carcinoma. *Oncogene.* 2022;41(9):1269-80.
7. Ewing B, Hillier L, Wendl MC, and Green P. Base-calling of automated sequencer traces using phred. I. Accuracy assessment. *Genome Res.* 1998;8(3):175-85.
8. Ewing B, and Green P. Base-calling of automated sequencer traces using phred. II. Error probabilities. *Genome Res.* 1998;8(3):186-94.
9. Edgar RC, Haas BJ, Clemente JC, Quince C, and Knight R. UCHIME improves sensitivity and speed of chimera detection. *Bioinformatics.* 2011;27(16):2194-200.
10. Schloss PD, and Westcott SL. Assessing and improving methods used in operational taxonomic unit-based approaches for 16S rRNA gene sequence analysis. *Appl Environ Microbiol.* 2011;77(10):3219-26.
11. Pruesse E, Peplies J, and Glockner FO. SINA: accurate high-throughput multiple sequence alignment of ribosomal RNA genes. *Bioinformatics.* 2012;28(14):1823-9.
12. Quast C, Pruesse E, Yilmaz P, Gerken J, Schweer T, Yarza P, et al. The SILVA ribosomal RNA gene database project: improved data processing and web-based tools. *Nucleic Acids Res.* 2013;41(Database issue):D590-6.
13. Team RC. *R: A Language and Environment for Statistical Computing, Vienna, Austria.* Vienna, Austria: R Foundation for Statistical Computing; 2019.
14. Robertson CE, Harris JK, Wagner BD, Granger D, Browne K, Tatem B, et al. Explicet: graphical user interface software for metadata-driven management, analysis and visualization of microbiome data. *Bioinformatics.* 2013;*29*(23):3100-1.
15. Anderson MJ, Crist TO, Chase JM, Vellend M, Inouye BD, Freestone AL, et al. Navigating the multiple meanings of beta diversity: a roadmap for the practicing ecologist. *Ecol Lett.* 2011;14(1):19-28.
16. Oksanen J, Blanchet G, Friendly M, Kindt R, Legendre P, McGlinn D, et al.; 2019.
17. Fernandes AD, Macklaim JM, Linn TG, Reid G, and Gloor GB. ANOVA-like differential expression (ALDEx) analysis for mixed population RNA-Seq. *PloS one.* 2013;8(7):e67019.
18. Fernandes AD, Reid JN, Macklaim JM, McMurrough TA, Edgell DR, and Gloor GB. Unifying the analysis of high-throughput sequencing datasets: characterizing RNA-seq, 16S rRNA gene sequencing and selective growth experiments by compositional data analysis. *Microbiome.* 2014;2:15.
19. Benjamini Y, and Hochberg Y. Controlling the false discovery rate: a practical and powerful approach to multiple testing. *J Roy Statist Soc Ser B.* 1995;57:289-300.
20. Lee JS, Wang RX, Goldberg MS, Clifford GP, Kao DJ, and Colgan SP. Microbiota-Sourced Purines Support Wound Healing and Mucous Barrier Function. *iScience.* 2020;23(6):101226.
21. Berlinberg AJ, Regner EH, Stahly A, Brar A, Reisz JA, Gerich ME, et al. Multi 'Omics Analysis of Intestinal Tissue in Ankylosing Spondylitis Identifies Alterations in the Tryptophan Metabolism Pathway. *Frontiers in Immunology.* 2021;12.
22. Banda NK, Kraus D, Vondracek A, Huynh LH, Bendele A, Holers VM, et al. Mechanisms of effects of complement inhibition in murine collagen-induced arthritis. *Arthritis & Rheumatism.* 2002;46(11):3065-75.
23. Kerwar SS, Englert ME, McReynolds RA, Jane Landes M, Lloyd JM, Oronsky AL, et al. Type ii collagen-induced arthritis Studies with Purified Anticollagen Immunoglobulin. *Arthritis & Rheumatism.* 1983;26(9):1120-31.

24. Chriswell ME, Lefferts AR, Clay MR, Hsu AR, Seifert J, Feser ML, et al. Clonal IgA and IgG autoantibodies from individuals at risk for rheumatoid arthritis identify an arthritogenic strain of <i>Subdoligranulum</i>. *Science Translational Medicine.* 2022;14(668):eabn5166.
25. Dillon SM, Guo K, Castleman MJ, Santiago ML, and Wilson CC. Quantifying HIV-1-Mediated Gut CD4+ T Cell Death in the Lamina Propria Aggregate Culture (LPAC) Model. *Bio-protocol.* 2020;10(2):: e3486.
26. Dillon SM, Rogers LM, Howe R, Hostetler LA, Buhrman J, McCarter MD, et al. Human intestinal lamina propria CD1c+ dendritic cells display an activated phenotype at steady state and produce IL-23 in response to TLR7/8 stimulation. *J Immunol.* 2010;184(12):6612-21.
27. Howe R, Dillon S, Rogers L, McCarter M, Kelly C, Gonzalez R, et al. Evidence for dendritic cell-dependent CD4(+) T helper-1 type responses to commensal bacteria in normal human intestinal lamina propria. *Clin Immunol.* 2009;131(2):317-32.
28. Berlinberg AJ, Brar A, Stahly A, Gerich ME, Fennimore BP, Scott FI, et al. A Novel Approach toward Less Invasive Multiomics Gut Analyses: a Pilot Study. *Microbiol Spectr.* 2022;10(2):e0244621.
29. Huber W, Carey VJ, Gentleman R, Anders S, Carlson M, Carvalho BS, et al. Orchestrating high-throughput genomic analysis with Bioconductor. *Nature Methods.* 2015;12(2):115-21.
30. Love MI, Anders S, Kim V, and Huber W. RNA-Seq workflow: gene-level exploratory analysis and differential expression. *F1000Research.* 2015;4:1070.
31. Patro R, Duggal G, Love MI, Irizarry RA, and Kingsford C. Salmon provides fast and bias-aware quantification of transcript expression. *Nature Methods.* 2017;14(4):417-9.
32. Frankish A, Diekhans M, Ferreira AM, Johnson R, Jungreis I, Loveland J, et al. GENCODE reference annotation for the human and mouse genomes. *Nucleic Acids Res.* 2019;47(D1):D766-d73.
33. Love MI, Soneson C, Hickey PF, Johnson LK, Pierce NT, Shepherd L, et al. Tximeta: Reference sequence checksums for provenance identification in RNA-seq. *PLOS Computational Biology.* 2020;16(2):e1007664.
34. Love MI, Huber W, and Anders S. Moderated estimation of fold change and dispersion for RNA-seq data with DESeq2. *Genome Biology.* 2014;15(12).

Supplemental Table 1.

Dataset: LPMC CD19+ B cells

UDP-N-acetyl-D-glucosamine Biosynthesis II
Pathway hits: 4/6, z-score: 2, p-value: 1.88E-04

Symbol	Entrez Gene Name	Ensembl	Expr Intensity/RPKM/FPKM/Counts	Expr Log Ratio	Expr p-value	Expr FDR (q-value)	Expr Fold Change	Expected	Location	Type(s)
GFPT1	glutamine--fructose-6-phosphate transaminase 1	ENSG00000198380.13	644.189	0.502	0.33	1	1.416	Up	Cytoplasm	enzyme
GNPNAT1	glucosamine-phosphate N-acetyltransferase 1	ENSG00000100522.10	63.444	0.717	0.319	1	1.644	Up	Cytoplasm	enzyme
PGM3	phosphoglucomutase 3	ENSG00000013375.16	179.912	0.558	0.4	1	1.472	Up	Cytoplasm	enzyme
UAP1	UDP-N-acetylglucosamine pyrophosphorylase 1	ENSG00000117143.13	284.406	0.539	0.327	1	1.452	Up	Nucleus	enzyme

Unfolded Protein Response
Pathway hits: 14/90, z-score: 2.121, p-value: 1.07E-03

Symbol	Entrez Gene Name	Ensembl	Expr Intensity/RPKM/FPKM/Counts	Expr Log Ratio	Expr p-value	Expr FDR (q-value)	Expr Fold Change	Expected	Location	Type(s)
DNAJB1	DnaJ heat shock protein family (Hsp40) member B1	ENSG00000132002.9	4872.28	0.509	0.469	1	1.423		Nucleus	transcription regulator
DNAJB13	DnaJ heat shock protein family (Hsp40) member B13	ENSG00000187726.9	15.777	0.681	0.054	1	1.603		Cytoplasm	other
DNAJC1	DnaJ heat shock protein family (Hsp40) member C1	ENSG00000136770.11	223.347	0.714	0.347	1	1.641		Cytoplasm	other
DNAJC3	DnaJ heat shock protein family (Hsp40) member C3	ENSG00000102580.15	615.599	0.514	0.484	1	1.428	Down	Cytoplasm	other
DNAJC6	DnaJ heat shock protein family (Hsp40) member C6	ENSG00000116675.16	65.824	0.549	0.099	1	1.463		Cytoplasm	other
DNAJC16	DnaJ heat shock protein family (Hsp40) member C16	ENSG00000116138.13	184.32	0.51	0.04	1	1.424		Cytoplasm	other
ERO1B	endoplasmic reticulum oxidoreductase 1 beta	ENSG00000086619.14	140.026	0.51	0.377	1	1.424	Up	Cytoplasm	enzyme
HSPA5	heat shock protein family A (Hsp70) member 5	ENSG00000044574.9	5794.735	0.529	0.478	1	1.443	Up	Cytoplasm	enzyme
HSPA6	heat shock protein family A (Hsp70) member 6	ENSG00000173110.8	1338.989	1.191	0.121	1	2.283	Up	Nucleus	enzyme
HSPA1A/H	heat shock protein family A (Hsp70) member 1A	ENSG00000204388.7	11949.356	0.845	0.298	1	1.796	Up	Cytoplasm	enzyme
MAPK8	mitogen-activated protein kinase 8	ENSG00000107643.16	221.591	0.606	0.157	1	1.522	Up	Cytoplasm	kinase
OS9	OS9 endoplasmic reticulum lectin	ENSG00000135506.16	795.047	0.55	0.285	1	1.464		Nucleus	other
SEL1L	SEL1L adaptor subunit of ERAD E3 ubiquitin ligase	ENSG00000071537.14	1055.271	0.748	0.264	1	1.68	Up	Cytoplasm	other
XBP1	X-box binding protein 1	ENSG00000100219.16	1737.376	0.743	0.329	1	1.674	Up	Nucleus	transcription regulator

NRF2-mediated oxidative stress response
Pathway hits: 27/237, z-score: 2.496, p-value: 1.35E-03

Symbol	Entrez Gene Name	Ensembl	Expr Intensity/RPKM/FPKM/Counts	Expr Log Ratio	Expr p-value	Expr FDR (q-value)	Expr Fold Change	Expected	Location	Type(s)
CBR1	carbonyl reductase 1	ENSG00000159228.13	38.027	0.616	0.185	1	1.533	Up	Cytoplasm	enzyme
CYP2C8	cytochrome P450 family 2 subfamily C member 8	ENSG00000138115.15	13.843	0.618	0.12	1	1.535		Cytoplasm	enzyme
CYP3A43	cytochrome P450 family 3 subfamily A member 43	ENSG00000021461.17	19.533	0.607	0.195	1	1.523		Cytoplasm	enzyme
DNAJB1	DnaJ heat shock protein family (Hsp40) member B1	ENSG00000132002.9	4872.28	0.509	0.469	1	1.423		Nucleus	transcription regulator
DNAJB13	DnaJ heat shock protein family (Hsp40) member B13	ENSG00000187726.9	15.777	0.681	0.054	1	1.603		Cytoplasm	other
DNAJC1	DnaJ heat shock protein family (Hsp40) member C1	ENSG00000136770.11	223.347	0.714	0.347	1	1.641		Cytoplasm	other
DNAJC3	DnaJ heat shock protein family (Hsp40) member C3	ENSG00000102580.15	615.599	0.514	0.484	1	1.428		Cytoplasm	other
DNAJC6	DnaJ heat shock protein family (Hsp40) member C6	ENSG00000116675.16	65.824	0.549	0.099	1	1.463		Cytoplasm	other
DNAJC16	DnaJ heat shock protein family (Hsp40) member C16	ENSG00000116138.13	184.32	0.51	0.04	1	1.424		Cytoplasm	other
ENC1	ectodermal-neural cortex 1	ENSG00000171617.15	107.579	0.584	0.061	1	1.499		Nucleus	peptidase
FOSL1	FOS like 1, AP-1 transcription factor subunit	ENSG00000175592.9	54.077	0.556	0.337	1	1.47	Down	Nucleus	transcription regulator
GSTA5	glutathione S-transferase alpha 5	ENSG00000182793.12	1.728	0.588	0.512	1	1.503		Cytoplasm	enzyme
GSTM1	glutathione S-transferase mu 1	ENSG00000134184.13	9.224	0.659	0.579	1	1.579		Cytoplasm	enzyme
HERPUD1	homocysteine inducible ER protein with ubiquitin like d	ENSG00000051108.15	2285.948	0.644	0.246	1	1.562	Up	Cytoplasm	other
HMOX1	heme oxygenase 1	ENSG00000100292.18	90.956	1.109	0.031	1	2.157	Up	Cytoplasm	enzyme
KEAP1	kelch like ECH associated protein 1	ENSG00000079999.14	133.896	0.539	0.199	1	1.453	Down	Cytoplasm	other
MAP2K3	mitogen-activated protein kinase kinase 3	ENSG00000034152.19	696.901	0.677	0.168	1	1.599	Up	Cytoplasm	kinase
MAPK8	mitogen-activated protein kinase 8	ENSG00000107643.16	221.591	0.606	0.157	1	1.522	Up	Cytoplasm	kinase
PIK3CG	phosphatidylinositol-4,5-bisphosphate 3-kinase catalyt	ENSG00000105851.11	770.839	0.868	0.144	1	1.825		Cytoplasm	kinase
PRKD1	protein kinase D1	ENSG00000184304.16	22.264	0.592	0.096	1	1.508	Up	Cytoplasm	kinase
RASD1	ras related dexamethasone induced 1	ENSG00000108551.5	63.158	1.337	0.094	1	2.526		Cytoplasm	enzyme
RRAS	RAS related	ENSG00000126458.4	8.697	0.598	0.249	1	1.514	Up	Cytoplasm	enzyme
SOD3	superoxide dismutase 3	ENSG00000109610.6	7.05	0.527	0.26	1	1.441		Extracellular	enzyme
SQSTM1	sequestosome 1	ENSG00000161011.20	3753.156	0.523	0.328	1	1.437	Up	Cytoplasm	transcription regulator
TXNRD1	thioredoxin reductase 1	ENSG00000198431.16	596.526	0.923	0.168	1	1.897	Up	Cytoplasm	enzyme
UBE2K	ubiquitin conjugating enzyme E2 K	ENSG00000078140.14	165.764	0.552	0.245	1	1.466	Up	Cytoplasm	transcription regulator
USP14	ubiquitin specific peptidase 14	ENSG00000101557.15	262.135	0.544	0.309	1	1.458	Up	Cytoplasm	peptidase

p38 MAPK signaling

Pathway hits: 15/118, z-score: 3.742, p-value: 5.47E-03

Symbol	Entrez Gene Name	Ensembl	Expr Intensity/RPKM/FPKM/Counts	Expr Log Ratio	Expr p-value	Expr FDR (q-value)	Expr Fold Change	Expected	Location	Type(s)
ATF1	activating transcription factor 1	ENSG00000123268.9	87.508	0.568	0.319	1	1.482	Up	Nucleus	transcription regulator
BORCS8-M	BORCS8-MEF2B readthrough	ENSG00000064489.23	13.878	0.896	0.558	1	1.861	Up	Nucleus	transcription regulator
FADD	Fas associated via death domain	ENSG00000168040.5	99.837	0.553	0.168	1	1.467		Cytoplasm	other
H3-4	H3.4 histone, cluster member	ENSG00000168148.4	2.848	0.555	0.558	1	1.469	Up	Nucleus	other
H3-3A/H3-	H3.3 histone A	ENSG00000132475.10	2802.453	0.724	0.162	1	1.652	Up	Nucleus	other
HMGN1	high mobility group nucleosome binding domain 1	ENSG00000205581.11	574.47	0.561	0.258	1	1.475	Up	Nucleus	transcription regulator
IL37	interleukin 37	ENSG00000125571.10	1.874	0.5	0.617	1	1.414	Up	Extracellular	cytokine
IRAK4	interleukin 1 receptor associated kinase 4	ENSG00000198001.14	110.971	0.687	0.014	1	1.61	Up	Cytoplasm	kinase
MAP2K3	mitogen-activated protein kinase kinase 3	ENSG00000034152.19	696.901	0.677	0.168	1	1.599	Up	Cytoplasm	kinase
MEF2B	myocyte enhancer factor 2B	ENSG00000213999.17	38.523	1.149	0.027	1	2.218	Up	Nucleus	transcription regulator
MEF2D	myocyte enhancer factor 2D	ENSG00000116604.18	1427.047	0.513	0.249	1	1.427	Up	Nucleus	transcription regulator
PLA2G4B	phospholipase A2 group IVB	ENSG00000243708.11	24.702	0.909	0.064	1	1.877	Up	Cytoplasm	enzyme
RPS6KA4	ribosomal protein S6 kinase A4	ENSG00000162302.13	101.57	0.537	0.189	1	1.451	Up	Cytoplasm	kinase
RPS6KA5	ribosomal protein S6 kinase A5	ENSG00000100784.12	795.016	0.527	0.003	1	1.44	Up	Nucleus	kinase
TAB2	TGF-beta activated kinase 1 (MAP3K7) binding protein	ENSG00000228408.6	38.394	0.511	0.342	1	1.425	Up	Cytoplasm	other

p53 signaling

Pathway hits: 13/98, z-score: 2.121, p-value: 6.55E-03

Symbol	Entrez Gene Name	Ensembl	Expr Intensity/RPKM/FPKM/Counts	Expr Log Ratio	Expr p-value	Expr FDR (q-value)	Expr Fold Change	Expected	Location	Type(s)
CASP6	caspase 6	ENSG00000138794.10	28.708	0.581	0.096	1	1.496	Up	Cytoplasm	peptidase
CDKN1A	cyclin dependent kinase inhibitor 1A	ENSG00000124762.14	1191.713	0.598	0.266	1	1.514	Up	Nucleus	kinase
GADD45A	growth arrest and DNA damage inducible alpha	ENSG00000116717.13	682.285	0.944	0.31	1	1.924		Nucleus	other
GADD45B	growth arrest and DNA damage inducible beta	ENSG00000099860.9	1537.727	0.53	0.385	1	1.444		Cytoplasm	other
GML	glycosylphosphatidylinositol anchored molecule like	ENSG00000104499.7	0.639	1.197	0.549	1	2.293	Up	Plasma Mem	other
KAT2B	lysine acetyltransferase 2B	ENSG00000114166.8	303.612	0.774	0.127	1	1.71	Up	Nucleus	transcription regulator
MAPK8	mitogen-activated protein kinase 8	ENSG00000107643.16	221.591	0.606	0.157	1	1.522	Up	Cytoplasm	kinase
PCNA	proliferating cell nuclear antigen	ENSG00000132646.11	98.69	0.868	0.027	1	1.825		Nucleus	enzyme
PIK3CG	phosphatidylinositol-4,5-bisphosphate 3-kinase catalyt	ENSG00000105851.11	770.839	0.868	0.144	1	1.825	Down	Cytoplasm	kinase
PPP1R13B	protein phosphatase 1 regulatory subunit 13B	ENSG00000088808.18	296.256	0.533	0.127	1	1.447		Cytoplasm	phosphatase
TIGAR	TP53 induced glycolysis regulatory phosphatase	ENSG00000078237.7	248.574	2.033	0.008	1	4.092		Cytoplasm	enzyme
TP73	tumor protein p73	ENSG00000078900.15	75.413	0.62	0.148	1	1.537	Up	Nucleus	transcription regulator
TP53INP1	tumor protein p53 inducible nuclear protein 1	ENSG00000164938.14	742.269	0.586	0.269	1	1.501	Up	Nucleus	other

HIF1α signaling

Pathway hits: 22/208, z-score: 3.273, p-value: 8.56E-03

Symbol	Entrez Gene Name	Ensembl	Expr Intensity/RPKM/FPKM/Counts	Expr Log Ratio	Expr p-value	Expr FDR (q-value)	Expr Fold Change	Expected	Location	Type(s)
ADRA1B	adrenoceptor alpha 1B	ENSG00000170214.5	15.618	0.749	0.136	1	1.681	Up	Plasma Mem	G-protein coupled recep
BMP6	bone morphogenetic protein 6	ENSG00000153162.9	271.571	0.603	0.533	1	1.519	Up	Extracellular	growth factor
CDKN1A	cyclin dependent kinase inhibitor 1A	ENSG00000124762.14	1191.713	0.598	0.266	1	1.514	Up	Nucleus	kinase
HMOX1	heme oxygenase 1	ENSG00000100292.18	90.956	1.109	0.031	1	2.157	Up	Cytoplasm	enzyme
HSPA5	heat shock protein family A (Hsp70) member 5	ENSG00000044574.9	5794.735	0.529	0.478	1	1.443	Down	Cytoplasm	enzyme
HSPA6	heat shock protein family A (Hsp70) member 6	ENSG00000173110.8	1338.989	1.191	0.121	1	2.283	Down	Nucleus	enzyme
HSPA1A/H	heat shock protein family A (Hsp70) member 1A	ENSG00000204388.7	11949.356	0.845	0.298	1	1.796	Down	Cytoplasm	enzyme
IL6R	interleukin 6 receptor	ENSG00000160712.13	445.117	0.872	0.232	1	1.83	Up	Plasma Mem	transmembrane recepto
LDHB	lactate dehydrogenase B	ENSG00000111716.14	188.01	0.548	0.224	1	1.462	Up	Cytoplasm	enzyme
MAP2K3	mitogen-activated protein kinase kinase 3	ENSG00000034152.19	696.901	0.677	0.168	1	1.599	Up	Cytoplasm	kinase
MMP1	matrix metallopeptidase 1	ENSG00000196611.6	15.476	0.559	0.069	1	1.473	Up	Extracellular	peptidase
MMP10	matrix metallopeptidase 10	ENSG00000166670.10	1.989	1.157	0.167	1	2.229	Up	Extracellular	peptidase
MMP15	matrix metallopeptidase 15	ENSG00000102996.5	37.677	0.876	0.009	1	1.836	Up	Extracellular	peptidase
MMP25	matrix metallopeptidase 25	ENSG00000008516.18	18.581	0.616	0.22	1	1.532	Up	Plasma Mem	peptidase
MMP28	matrix metallopeptidase 28	ENSG00000271447.6	17.636	0.64	0.084	1	1.558	Up	Extracellular	peptidase
PIK3CG	phosphatidylinositol-4,5-bisphosphate 3-kinase catalyt	ENSG00000105851.11	770.839	0.868	0.144	1	1.825	Up	Cytoplasm	kinase
PRKD1	protein kinase D1	ENSG00000184304.16	22.264	0.592	0.096	1	1.508	Up	Cytoplasm	kinase
PROK1	prokineticin 1	ENSG00000143125.6	4.916	0.641	0.228	1	1.56	Up	Extracellular	growth factor
RAC3	Rac family small GTPase 3	ENSG00000169750.9	2.296	0.764	0.432	1	1.698		Cytoplasm	enzyme
RASD1	ras related dexamethasone induced 1	ENSG00000108551.5	63.158	1.337	0.094	1	2.526	Up	Cytoplasm	enzyme
RRAS	RAS related	ENSG00000126458.4	8.697	0.598	0.249	1	1.514	Up	Cytoplasm	enzyme
SLC2A1	solute carrier family 2 member 1	ENSG00000117394.24	505.117	0.632	0.125	1	1.549	Up	Plasma Mem	transporter

IL-17 signaling<br>Pathway hits: 19/187, z-score: 4.359, p-value: 2.03E-02											
**Symbol**	**Entrez Gene Name**	**Ensembl**	**Expr Intensity/RPKM/FPKM/Counts**	**Expr Log Ratio**	**Expr p-value**	**Expr FDR (q-value)**	**Expr Fold Change**	**Expected**	**Location**	**Type(s)**	
CCL11	C-C motif chemokine ligand 11	ENSG00000172156.4	2.33	1.553	0.082	1	2.933	Up	Extracellular	cytokine	
CD40LG	CD40 ligand	ENSG00000102245.8	3.566	0.648	0.315	1	1.567	Up	Extracellular	cytokine	
CSF2	colony stimulating factor 2	ENSG00000164400.6	3.722	1.088	0.17	1	2.126	Up	Extracellular	cytokine	
DEFB119	defensin beta 119	ENSG00000180483.7	4.078	0.553	0.337	1	1.467	Up	Extracellular	other	
DEFB104A	defensin beta 104A	ENSG00000177023.2	1.792	0.762	0.433	1	1.695	Up	Extracellular	other	
DEFB107A	defensin beta 107A	ENSG00000186572.2	0.176	1.056	0.738	1	2.08	Up	Extracellular	other	
DEFB130A	defensin beta 130A	ENSG00000233050.1	0.401	1.041	0.556	1	2.057	Up	Extracellular	other	
DEFB4A/D	defensin beta 4A	ENSG00000171711.3	0.763	0.573	0.78	1	1.488	Up	Extracellular	other	
IL2	interleukin 2	ENSG00000109471.5	6.93	0.697	0.269	1	1.621	Up	Extracellular	cytokine	
IL3	interleukin 3	ENSG00000164399.5	2.863	0.817	0.24	1	1.762	Up	Extracellular	cytokine	
IL31	interleukin 31	ENSG00000204671.2	2.192	0.603	0.538	1	1.519	Up	Extracellular	other	
IL37	interleukin 37	ENSG00000125571.10	1.874	0.5	0.617	1	1.414	Up	Extracellular	cytokine	
MAP2K3	mitogen-activated protein kinase kinase 3	ENSG00000034152.19	696.901	0.677	0.168	1	1.599	Up	Cytoplasm	kinase	
MAPK8	mitogen-activated protein kinase 8	ENSG00000107643.16	221.591	0.606	0.157	1	1.522	Up	Cytoplasm	kinase	
PIK3CG	phosphatidylinositol-4,5-bisphosphate 3-kinase catalyt	ENSG00000105851.11	770.839	0.868	0.144	1	1.825	Up	Cytoplasm	kinase	
PROK1	prokineticin 1	ENSG00000143125.6	4.916	0.641	0.228	1	1.56	Up	Extracellular	growth factor	
RASD1	ras related dexamethasone induced 1	ENSG00000108551.5	63.158	1.337	0.094	1	2.526	Up	Cytoplasm	enzyme	
RRAS	RAS related	ENSG00000126458.4	8.697	0.598	0.249	1	1.514	Up	Cytoplasm	enzyme	
TAB2	TGF-beta activated kinase 1 (MAP3K7) binding protein	ENSG00000228408.6	38.394	0.511	0.342	1	1.425	Up	Cytoplasm	other	

Supplemental Table 2.

Dataset: LPMC CD3+ T cells										
**Differential regulation of cytokine production in intestinal epithelial cells by IL-17A/F**										
**Pathway hits: 5/23, z-score: 2.236, p-value: 2.82E-03**										
**Symbol**	**Entrez Gene Name**	**Ensembl**	**Expr Intensity/RPKM/FPKM/Counts**	**Expr Log Ratio**	**Expr p-value**	**Expr FDR (q-value)**	**Expr Fold Change**	**Expected**	**Location**	**Type(s)**
CCL4	C-C motif chemokine ligand 4	ENSG00000275302.2	100.812	0.656	0.303	1	1.575	Up	Extracellular Space	cytokine
DEFB1	defensin beta 1	ENSG00000164825.4	1.345	2.582	0.031	1	5.988	Up	Extracellular Space	other
DEFB4A/D	defensin beta 4A	ENSG00000177257.3	0.745	0.929	0.571	1	1.904	Up	Extracellular Space	other
IFNG	interferon gamma	ENSG00000111537.5	50.736	0.651	0.399	1	1.57	Up	Extracellular Space	cytokine
TNF	tumor necrosis factor	ENSG00000232810.4	131.681	0.519	0.286	1	1.433	Up	Extracellular Space	cytokine
**Neuroprotective Role of THOP1 in Alzheimer's Disease**										
**Pathway hits: 12/118, z-score: 2.714, p-value: 5.76E-03**										
**Symbol**	**Entrez Gene Name**	**Ensembl**	**Expr Intensity/RPKM/FPKM/Counts**	**Expr Log Ratio**	**Expr p-value**	**Expr FDR (q-value)**	**Expr Fold Change**	**Expected**	**Location**	**Type(s)**
ENDOU	endonuclease, poly(U) specific	ENSG00000111405.9	26.739	0.858	0.045	1	1.813	Up	Cytoplasm	peptidase
GNRH2	gonadotropin releasing hormone 2	ENSG00000125787.11	4.598	0.761	0.187	1	1.694	Down	Extracellular Space	other
HGFAC	HGF activator	ENSG00000109758.9	3.314	0.69	0.321	1	1.613	Up	Extracellular Space	peptidase
HPN	hepsin	ENSG00000105707.15	19.745	0.866	0.032	1	1.823	Up	Plasma Membrane	peptidase
HTRA1	HtrA serine peptidase 1	ENSG00000166033.13	12.827	0.712	0.182	1	1.638	Up	Extracellular Space	peptidase
IFNG	interferon gamma	ENSG00000111537.5	50.736	0.651	0.399	1	1.57	Up	Extracellular Space	cytokine
KLK1	kallikrein 1	ENSG00000167748.11	22.834	0.553	0.115	1	1.467	Up	Cytoplasm	peptidase
KLK8	kallikrein related peptidase 8	ENSG00000129455.15	12.375	0.508	0.164	1	1.422	Up	Extracellular Space	peptidase
NTS	neurotensin	ENSG00000133636.11	3.627	0.973	0.164	1	1.963		Extracellular Space	other
PRSS57	serine protease 57	ENSG00000185198.12	3.812	0.588	0.479	1	1.504	Up	Extracellular Space	peptidase
PRTN3	proteinase 3	ENSG00000196415.10	1.987	0.667	0.565	1	1.588	Up	Extracellular Space	peptidase
TMPRSS9	transmembrane serine protease 9	ENSG00000178297.14	25.678	0.683	0.031	1	1.606	Up	Plasma Membrane	peptidase
**IL-17 Signaling**										
**Pathway hits: 15/187, z-score: 3.873, p-value: 1.83E-02**										
**Symbol**	**Entrez Gene Name**	**Ensembl**	**Expr Intensity/RPKM/FPKM/Counts**	**Expr Log Ratio**	**Expr p-value**	**Expr FDR (q-value)**	**Expr Fold Change**	**Expected**	**Location**	**Type(s)**
CD40LG	CD40 ligand	ENSG00000102245.8	190.987	0.586	0.371	1	1.501	Up	Extracellular Space	cytokine
DEFB1	defensin beta 1	ENSG00000164825.4	1.345	2.582	0.031	1	5.988	Up	Extracellular Space	other
DEFB116	defensin beta 116	ENSG00000215545.1	0.849	1.125	0.489	1	2.182	Up	Extracellular Space	other
DEFB124	defensin beta 124	ENSG00000180383.4	13.152	0.542	0.17	1	1.456	Up	Extracellular Space	other
DEFB105A	defensin beta 105A	ENSG00000186562.8	7.298	0.56	0.248	1	1.474	Up	Extracellular Space	other
DEFB107A	defensin beta 107A	ENSG00000198129.3	0.748	0.843	0.62	1	1.794	Up	Extracellular Space	other
DEFB108B	defensin beta 108B	ENSG00000184276.3	2.1	1.188	0.166	1	2.279	Up	Extracellular Space	other
DEFB4A/D	defensin beta 4A	ENSG00000177257.3	0.745	0.929	0.571	1	1.904	Up	Extracellular Space	other
ERAS	ES cell expressed Ras	ENSG00000187682.2	2.042	0.784	0.515	1	1.722	Up	Plasma Membrane	enzyme
IFNG	interferon gamma	ENSG00000111537.5	50.736	0.651	0.399	1	1.57	Up	Extracellular Space	cytokine
MMP3	matrix metallopeptidase 3	ENSG00000149968.12	9.435	1.168	0.121	1	2.247	Up	Extracellular Space	peptidase
OSM	oncostatin M	ENSG00000099985.4	16.33	0.563	0.196	1	1.477	Up	Extracellular Space	cytokine
PIK3C2G	phosphatidylinositol-4-phosphate 3-	ENSG00000139144.11	28.442	0.517	0.205	1	1.43	Up	Cytoplasm	kinase
TNF	tumor necrosis factor	ENSG00000232810.4	131.681	0.519	0.286	1	1.433	Up	Extracellular Space	cytokine
TNFSF9	TNF superfamily member 9	ENSG00000125657.5	57.962	0.564	0.314	1	1.478	Up	Plasma Membrane	cytokine

Supplemental Table 3.

Antibody	Fluorophore	Clone	Vendor	Catalog #
CD4	BUV395	GK1.5	BD Bioscience	565974
Viability	Ghost Violet510		Cytek/Tonbo	13-0870-T100
CD44	BV605	IM7	Biolegend	103047
RORγt	BV786	Q31-378	BD Bioscience	564723
CD25	FITC	PC61.5	Cytek/Tonbo	35-0251-U025
CD3	PerCPCy5.5	145-2C11	Cytek/Tonbo	65-0031-U025
FoxP3	PE-Cy7	3G3	Cytek/Tonbo	60-5773-U025
CD62L	APC-Cy7	MEL-14	Biolegend	104427

Supplemental Table 4.

Antibody	Use	Vendor	Catalog Number
IgG Goat Anti-Mouse Complement C3	C3 IHC	MP Biomedicals	0855463
Goat-on-Rodent HRP Polymer & Probe	C3 IHC	BioCare Medical	GHP516H
Anti-mouse complement C3 goat IgG fraction, peroxidase conjugated	ELISA	MP Biomedicals	0855557
Goat-anti-mouse IgG Fab-HRP	ELISA	Southern Biotech	1015-05
Goat-anti-mouse IgG1-HRP	ELISA	Southern Biotech	1071-05
Goat-anti-mouse IgG2a	ELISA	Southern Biotech	1081-05
Goat-anti-mouse IgG2b	ELISA	Southern Biotech	1091-05

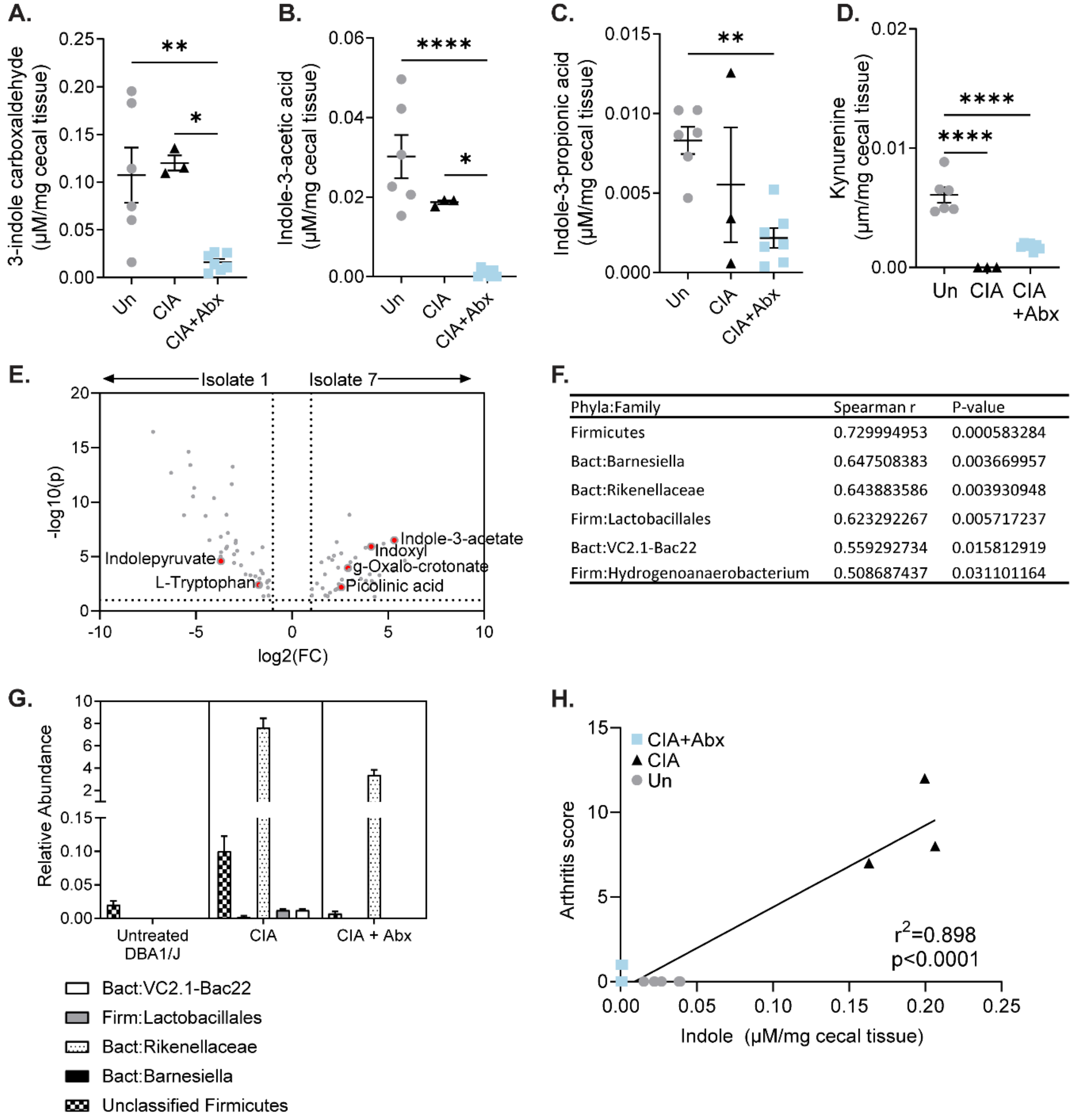

Phyla:Family	Spearman r	P-value
Firmicutes	0.729994953	0.000583284
Bact:Barnesiella	0.647508383	0.003669957
Bact:Rikenellaceae	0.643883586	0.003930948
Firm:Lactobacillales	0.623292267	0.005717237
Bact:VC2.1-Bac22	0.559292734	0.015812919
Firm:Hydrogenoanaerobacterium	0.508687437	0.031101164

Supplemental Figure 1. CIA induced microbiome-dependent changes in Trp metabolism. CIA was induced in male 6-week old DBA/1J mice by injection of bovine type II collagen (CII) in complete Freund's adjuvant at days 0 and 21. Cecal contents were harvested at day 35 from CIA mice (n=3-5), CIA mice depleted from microbiota by antibiotic administration after day 21 (CIA+Abx, n=7), or untreated DBA/1J mice (Un, n=6). **(A-D)** HPLC was used to quantify Trp pathway metabolites indicated on the y-axis in µm. All data were reported as individual mice (symbols) and mean ±SEM (bars) after normalization to weight (mg) of cecal contents. *, p<0.05; ***, p<0.001; ****, p<0.0001 as determined one-way ANOVA with Bonferroni correction for multiple comparisons. **(E)** Germ free DBA1 mice were orally gavaged at day 0 with either sterile PBS or 10^7 CFU *Subdoligranulum didolesgii Isolate 1 or Isolate 7.* LC-MS/MS were used to screen >190 metabolites in cecal contents. Differential abundance of metabolites is shown as a volcano plot. **(F)** Paired 16S amplicon sequencing + LC-MS/MS metabolomics analysis were performed on mice with CIA compared to CIA + Abx. Spearman correlation revealed the top 6 OTUs that significantly correlated with levels of indoxyl. N=5-7 per group. **(G)** Relative abundance of the OTUs identified in (F). **(H)** Indole was measured by HPLC in the cecal contents of untreated DBA/J mice and mice with CIA ± Abx. Pearson r correlation of arthritis severity vs indole levels is shown.

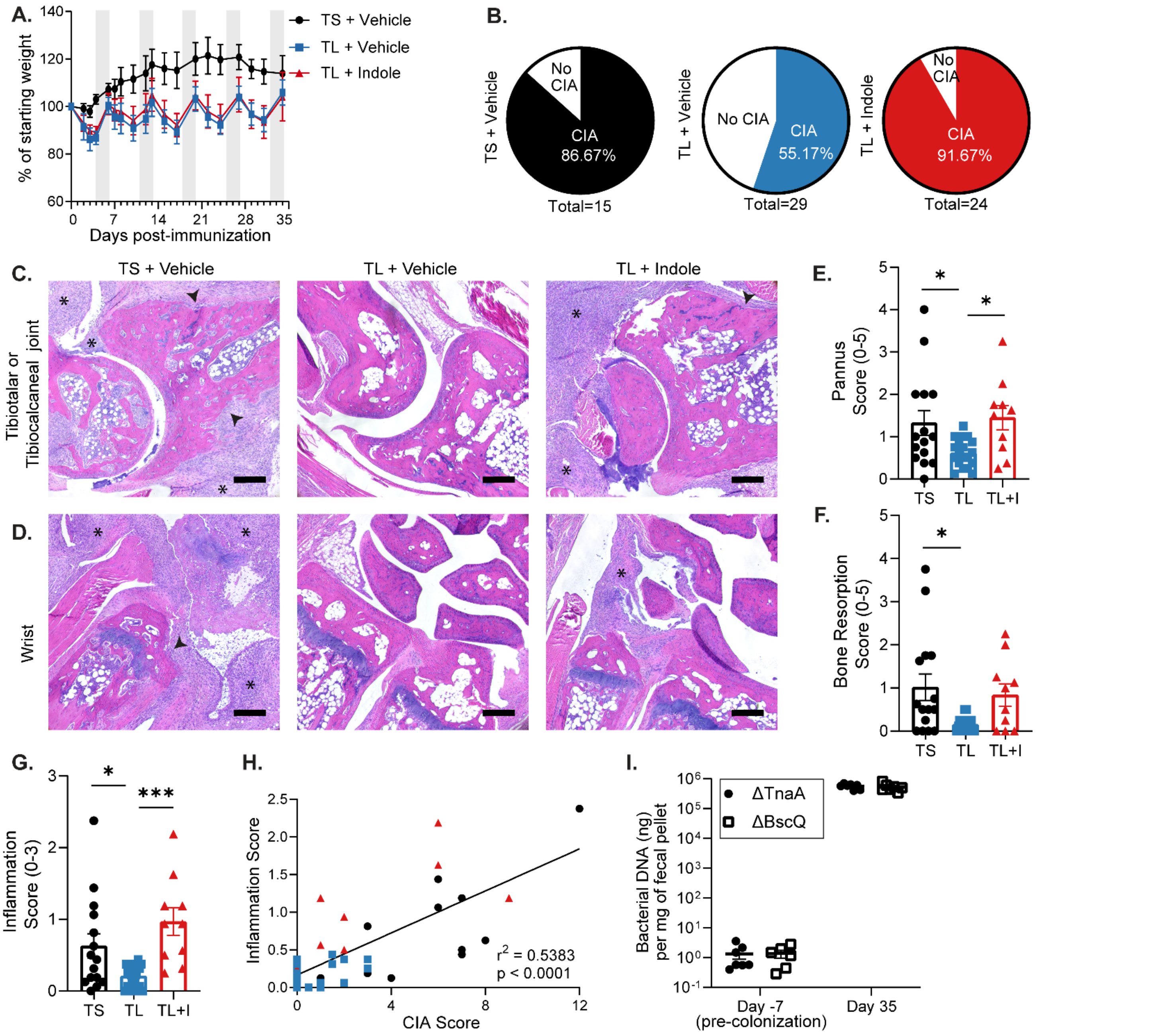

Supplemental Figure 2. Indole is required for CIA. Male 6-week old DBA/1J mice were fed a tryptophan-low (TL) diet or a trp-sufficient (TS) diet starting at day -1 through the duration of the experiment. CIA was induced by injection of CII in CFA at days 0 and 21. Indole (200µl of a 10mM solution) or vehicle control (0.33% methanol) was added back by oral gavage every other day starting on day 0. **(A)** % of starting body weight of mice with CIA on TL + vehicle, TL + indole, and TS + vehicle diets for the duration of the CIA study. In the TL treatment, after 5 days of Trp-deficient diet, mice are fed Trp-sufficient diet for 2 days (represented as grey bars) for a cumulative Trp-low diet. All values are plotted as mean ± SEM with n=10 (TL + vehicle), n=10 (TL + indole), n=5 (TS + vehicle) mice from one representative experiment. P<0.0001 by two-way ANOVA with Bonferroni correction for multiple comparisons for TL+Vehicle vs TS+Vehicle and TL+Indole vs TS+Vehicle. There was no statistical significance between TL+Vehicle and TL+Indole. **(B)** CIA incidence of mice in Figure 2B as defined by CIA score >1 at day 35, pooled from three independent experiments. **(C-D)** Representative H&E images of the tibiotalar joint **(C)** and wrist **(D)** for each group. Scale bar = 200µm; asterisks = synovial inflammation; arrowheads = bone resorption. **(E-G)** H&E stained paws were assessed for pannus formation (0-5), bone resorption (0-5), and inflammation (0-3), respectively. Each data point represents the average score of 4 paws per mouse. N=10-20 per group. *, p<0.05; **, p<0.01; ****, p<0.0001 as determined one-way ANOVA with Bonferroni correction for multiple comparisons. **(H)** Correlation between CIA score (clinical) and inflammation score (H&E). Pearson r^2 value and two-tailed p-value are shown. **(I)** Colonization of *E. coli* BW25113 *ΔtnaA* or *E.coli* BW25113 *ΔbcsQ*

in germ-free mice was verified by universal rpoB primers at CIA day 35 compared to pre-colonization (day-7). No significant differences were identified by unpaired t-test.

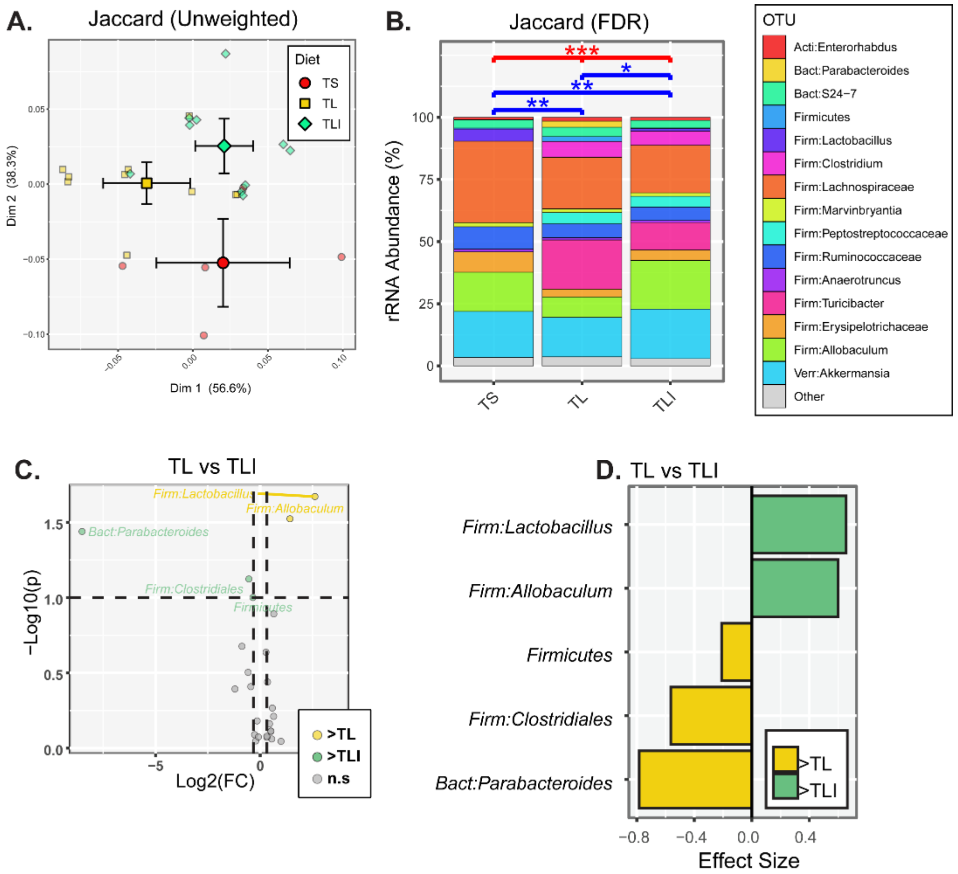

Supplemental Figure 3. Indole minimally affects bacterial dysbiosis imparted by a TL diet during CIA. The fecal microbiomes of mice in Figure 3 were also analyzed by Jaccard (unweighted) beta-diversity indices. **(A)** PCoA in which smaller, fainter symbols represent individual mice while larger symbols represent group means + 95% confidence intervals for PC1 and PC2. N=5-10 per group. **(B)** Bar charts annotated with results of PERMANOVA tests. *, p<0.05; **, p<0.01 and ***, p<0.001. **(C-D)** Volcano **(C)** and effect size **(D)** plots generated by ANOVA-like differential expression (ALDEx2) analysis indicate taxa that were enriched or depleted in TL + vehicle mice compared to TL + Indole. No taxa were found to be differentially abundant between TL + vehicle and TL + Indole groups after adjusting p-values for multiple comparisons (i.e., all FDR-corrected p values were >0.05), so p-value rather than FDR is shown in the volcano plot. N=5-10 per group.

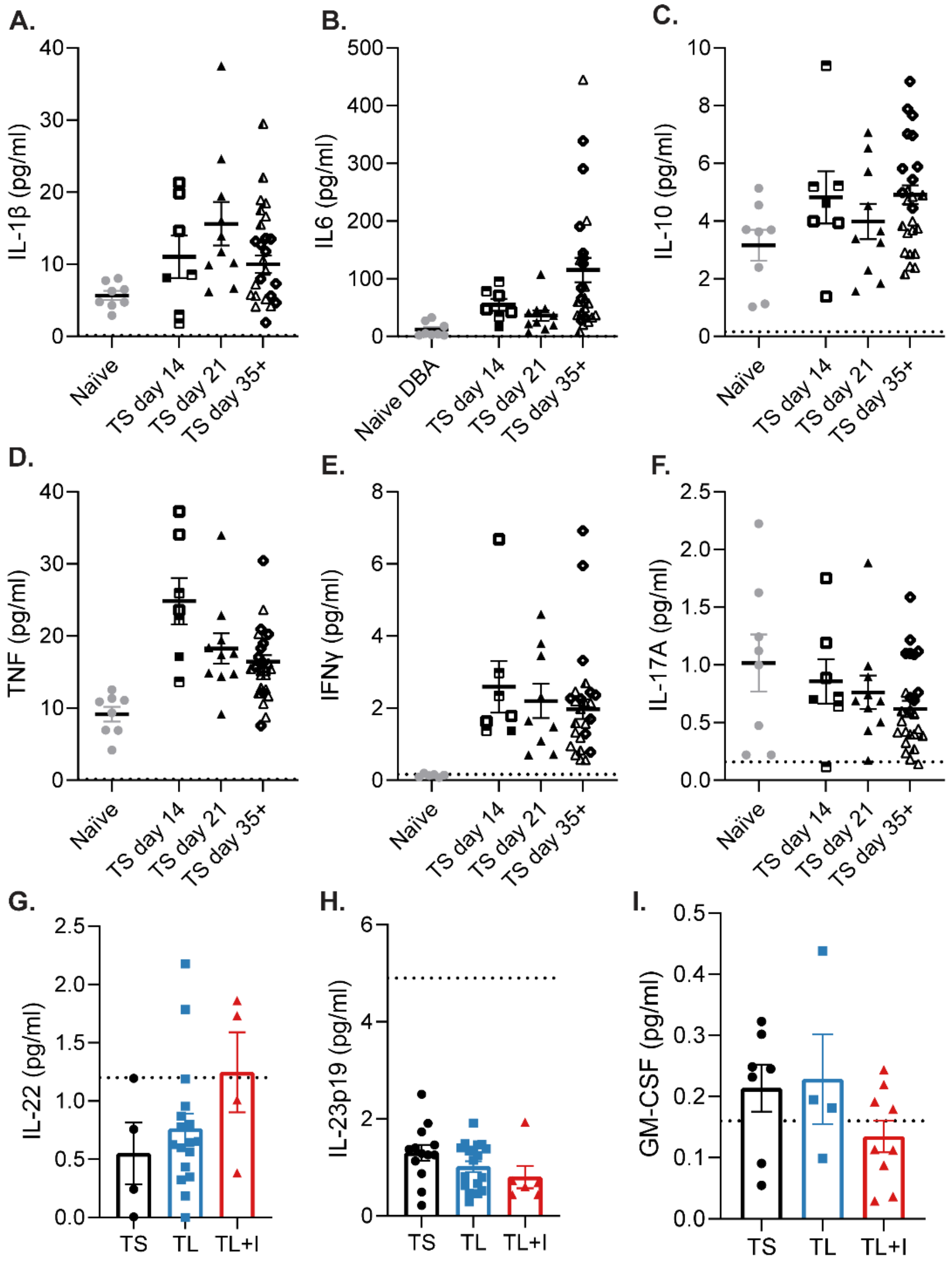

Supplemental Figure 4. Indole alters the cytokine profile in CIA. (A-F) Terminal serum was collected from male 6-week old DBA1 mice with CIA fed TS diet and treated with vehicle control (0.33% methanol) on CIA day 14, 21, and 35+. Naïve, age-matched, unimmunized male DBA1 mice were used as controls. Serum was analyzed by an 8-plex immunoassay (Mesoscale). The dashed line on the Y-axis denotes the lower limit of detection for each analyte. N=7-28 per timepoint plotted as individual mice (symbols) and mean ±SEM (bars). Each unique symbol represents an independent experiment. **(G-I)**, IL-22, IL-23p19, and GM-CSF were measured at day 35 only.

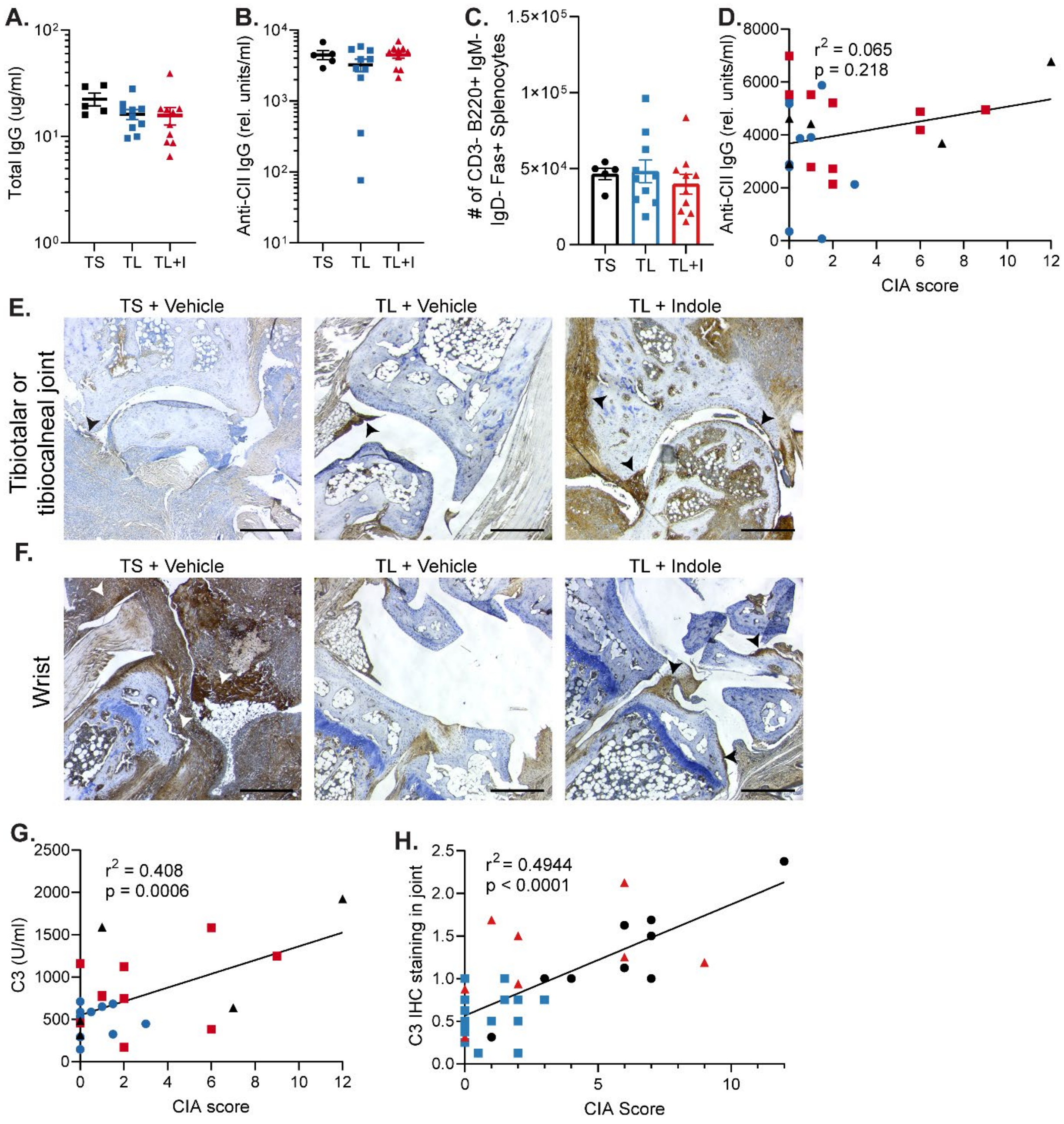

Supplemental Figure 5. Antibody isotype and complement fixation correlate with development of CIA. Day 35 serum from mice with CIA treated with TS + vehicle, TL + vehicle, and TL + indole was evaluated by ELISA for: **(A)** total IgG, **(B)** CII-specific IgG. **(C)** Splenocytes from mice with CIA fed a TL or TS diet and treated with indole or vehicle were counted and germinal center B cells (live CD3- B220+ IgM- IgD- Fas+) were assessed by flow cytometry. N=5-10 per group plotted as individual mice (symbols) and mean ±SEM (bars). No statistical significance was observed by one-way ANOVA with Bonferroni correction for multiple comparisons. **(D)** Anti-CII IgG levels were plotted against CIA score (x-axis). **(E-F)** Representative images of FFPE paws that were stained by immunohistochemistry for complement C3 (brown) and hematoxylin (blue). Scale bar = 200µm. Arrowheads = complement deposition. **(G)** CII-specific C3 activation was measured as described in Figure 5 and plotted against CIA severity. **(H)** C3 deposition by IHC was scored as described in figure 5 and plotted against CIA severity. Pearson r^2 value and two-tailed p-value are shown (panels D, G, H).

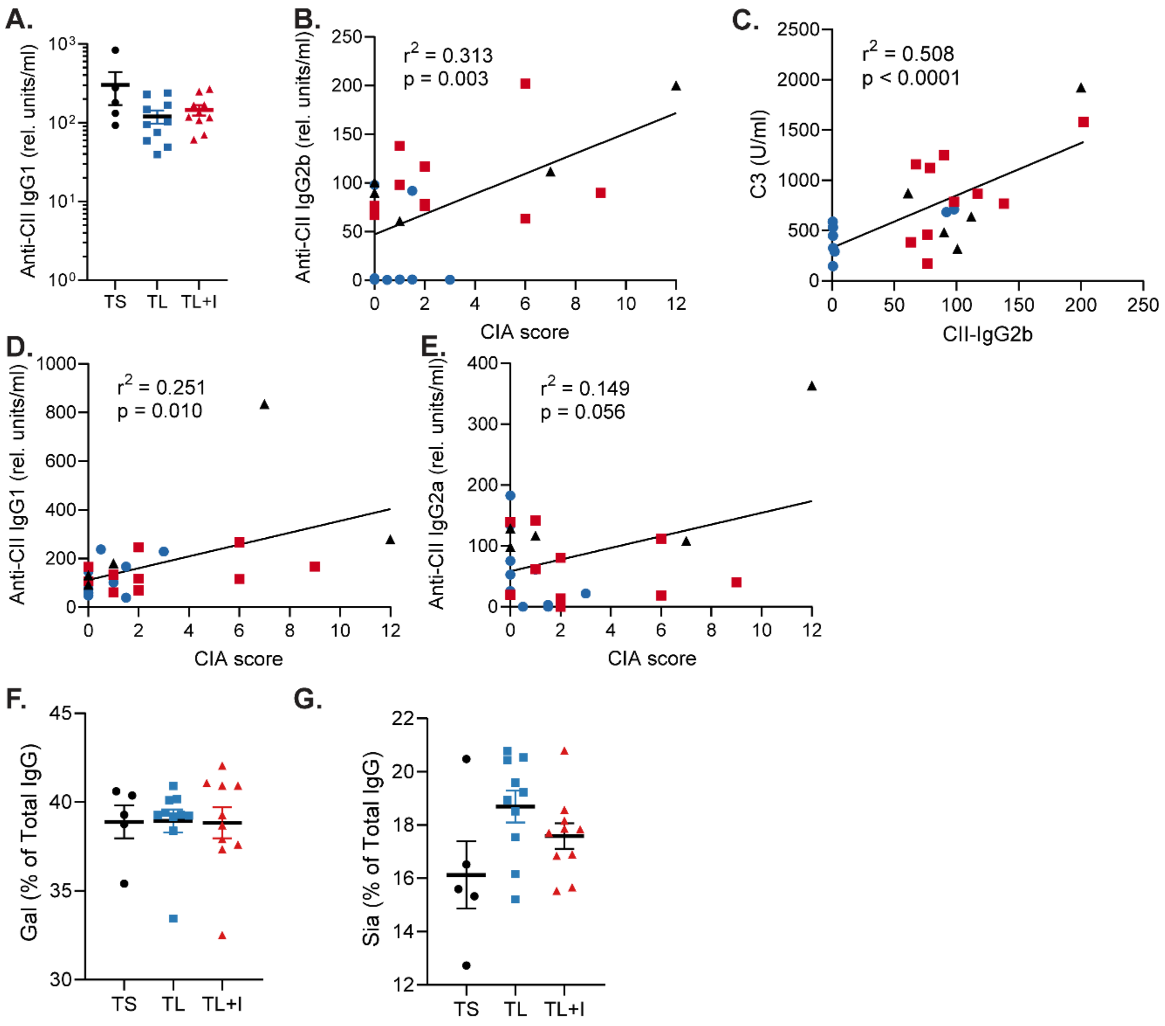

Supplemental Figure 6. Indole alters complement activation by CII-specific antibodies as well as IgG subclass and glycosylation. (A) Anti-CII IgG1 was measured in serum by ELISA at CIA day 35. **(B-C)** Anti-CII IgG2b was measured as described in Figure 5 and plotted against CIA score **(B)** and C3 activation **(C)**. **(D-E)** Anti-CII IgG1 and IgG2a were measured as described in Figure 5 and plotted against CIA score. **(F-G)** Total IgG was purified from serum and IgG glycosylation patterns were assessed by liquid chromatography with mass spectrometry (LC-MS/MS). Galactosylation and Sialylation were calculated as a % of all glycoforms (G0, G1, G2, S1, and S2). N=5-10 per group plotted as individual mice (symbols) and mean ±SEM (bars). No statistical significance was detected by one-way ANOVA with Bonferroni correction for multiple comparisons (panels A, F, G).). Pearson r^2 value and two-tailed p-value are shown (panels B-E).

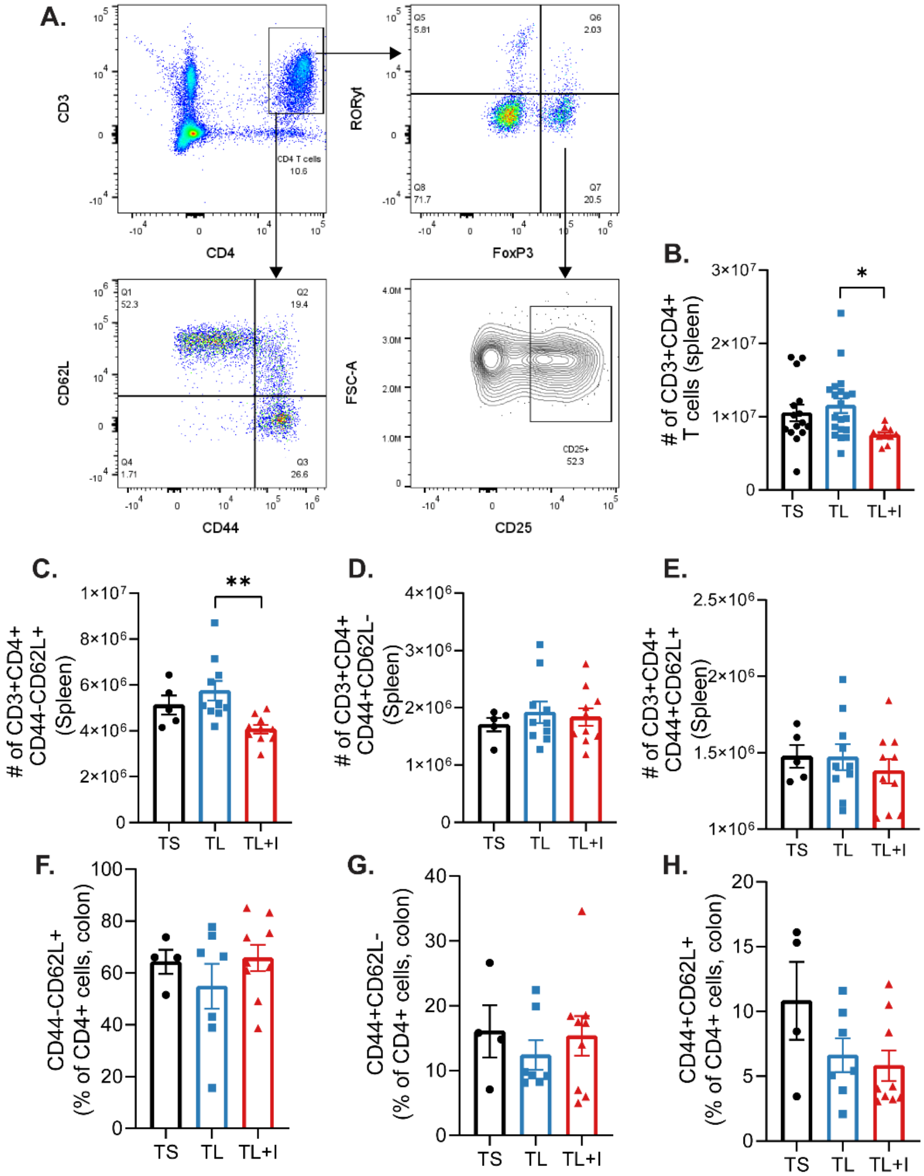

Supplemental Figure 7. Indole skews effector T cell populations. Splenocytes and colon LPMCs from mice with CIA fed a TL or TS diet and treated with indole or vehicle were analyzed by flow cytometry. **(A)** Representative gating strategy for Figure 6. **(B)** Total splenic CD3+CD4+ T cell counts at CIA day 35. N=10-20 per group pooled from two experiments. **(C)** Splenic $T_{naïve}$ (CD44-CD62L+) as # of CD4+ T cells. **(D)** Splenic $T_{effector}$ (CD44+CD62L-) as # of CD4+ T cells. **(E)** Splenic T_{CM} (CD44+CD62L+) as # of CD4+ T cells. **(F)** Colon $T_{naïve}$ (CD44-CD62L+) as % of CD4+ T cells. **(G)** Colon $T_{effector}$ (CD44+CD62L-) as % of CD4+ T cells. **(H)** Colon T_{CM} (CD44+CD62L+) as % of CD4+ T cells. N= 4-10 per group from one experiment plotted as individual mice (symbols) and mean ± SEM (bars). *, $p<0.05$; **, $p<0.01$ by one-way ANOVA with Bonferroni correction for multiple comparisons.

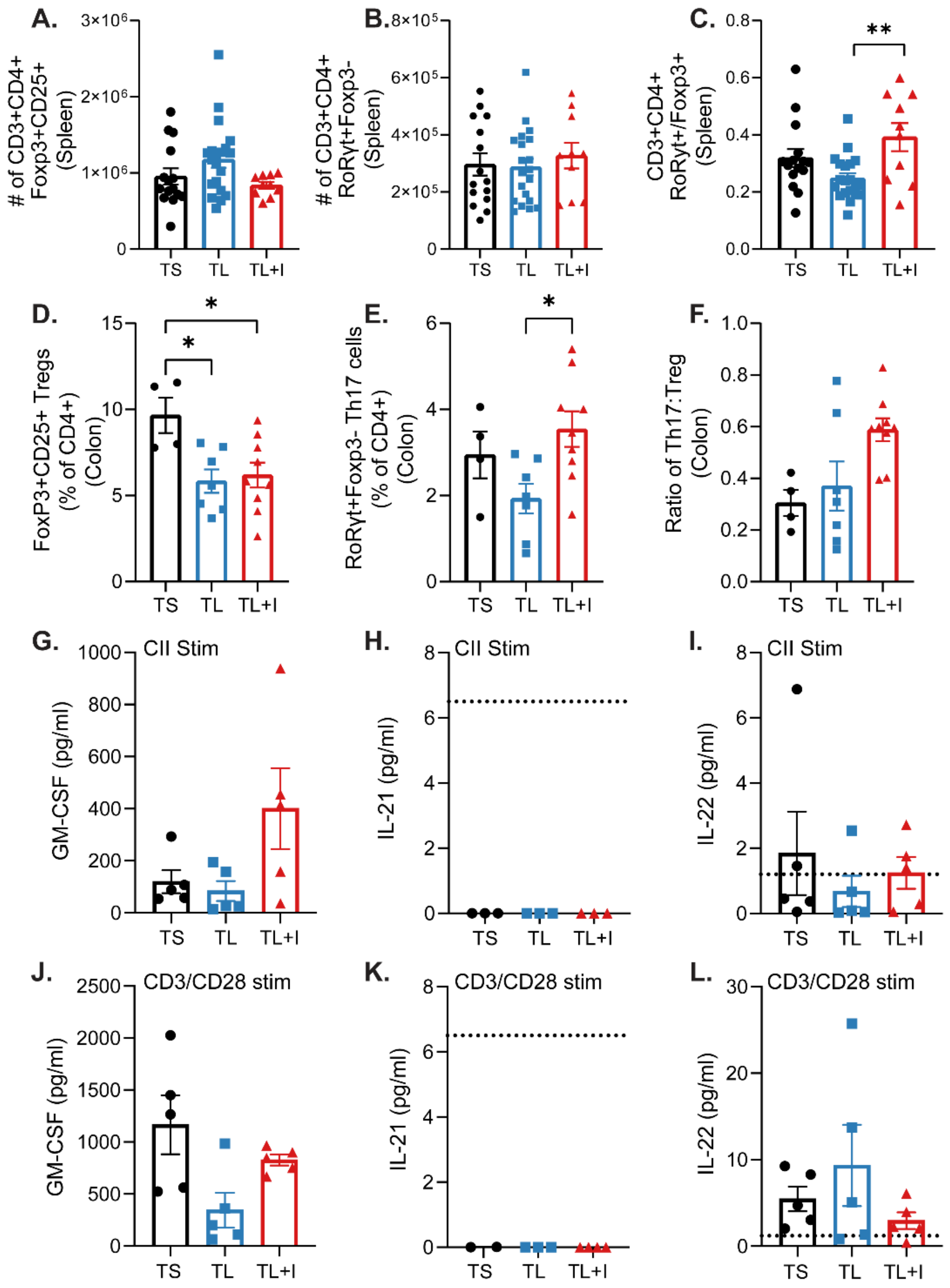

Supplemental Figure 8. Indole skews towards Th17 cells. (A) Total # of splenic CD3+CD4+FoxP3+RORγt- Treg cells **(B)** Total # of splenic FoxP3-RORγt+Th17 cells. **(C)** Ratio of the # of splenic Th17 cells:Treg cells. **(D)** Colon FoxP3+RORγt-CD25+ regulatory T cells are plotted as the percent of total CD4+ T cells. **(E)** Colon CD3+CD4+FoxP3-RORγt+ Th17 cells are plotted as the percent of total CD4+ T cells. **(F)** Ratio of colon Th17 to Treg cells. N=10-20 per group (spleen) and 4-10 per group (colon) plotted as individual mice (symbols) and mean ± SEM (bars). **(G-L)** Total splenocytes from CIA day 35 were harvested and re-stimulated with bovine type II collagen **(G-I)** or CD3/CD28 Dynabeads **(J-L)**; supernatant was saved and cytokines (as denoted on the y-axis) were measured by MSD**.** N=5 per group from one representative experiment. All data plotted as individual mice (symbols) and mean ± SEM (bars) with *, $p<0.05$; **, $p<0.01$ by one-way ANOVA with Bonferroni correction for multiple comparisons.

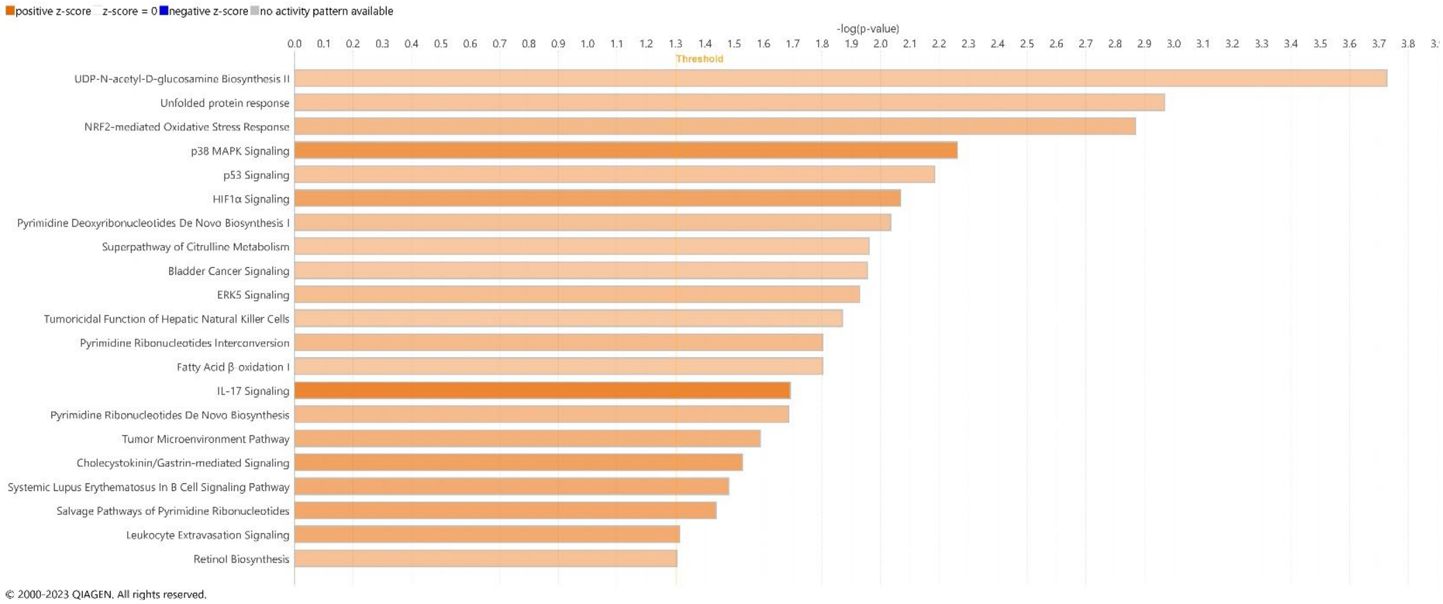

Supplemental Figure 9. Indole upregulates transcriptional pathways in human colon B cells. LPMCs from healthy human colon tissue were stimulated with 1 mM indole or vehicle for 4hr followed by RNA was isolated from flow-sorted CD19+ B cells for RNAseq. Differentially expressed pathways (indole vs vehicle) were identified with Ingenuity Pathway Analysis for CD19+ B cells. N=5 paired samples.

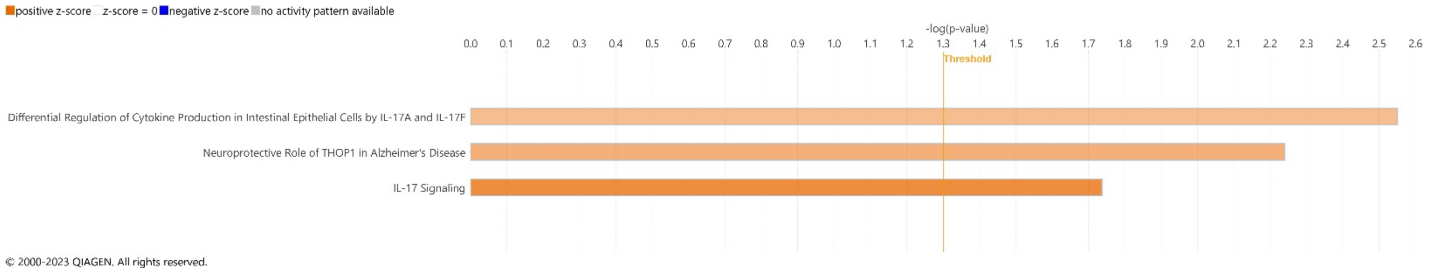

Supplemental Figure 10. Indole upregulates transcriptional pathways in human colon T cells. LPMCs from healthy human colon tissue were stimulated with 1 mM indole or vehicle for 4hr followed by RNA was isolated from flow-sorted CD3+ T cells for RNAseq. Differentially expressed pathways (indole vs vehicle) were identified with Ingenuity Pathway Analysis for CD3+ T cells. N=5 paired samples.
